# Supplementary material for: Learning to detect AI texts and learning the limits
Source: PLoS One. 2025 Oct 15;20(10):e0333007. doi: 10.1371/journal.pone.0333007 (PMC12527182; doi:10.1371/journal.pone.0333007)
Supplement: S2 File — All data and scripts used in the study, some additional descriptive statistics, scalable formate figures. (ZIP) [file pone.0333007.s002.tgz › data and scripts/OSF/scripts/analysis/Demographics/readability_Demographics-ai_lacks_creativity.pdf]

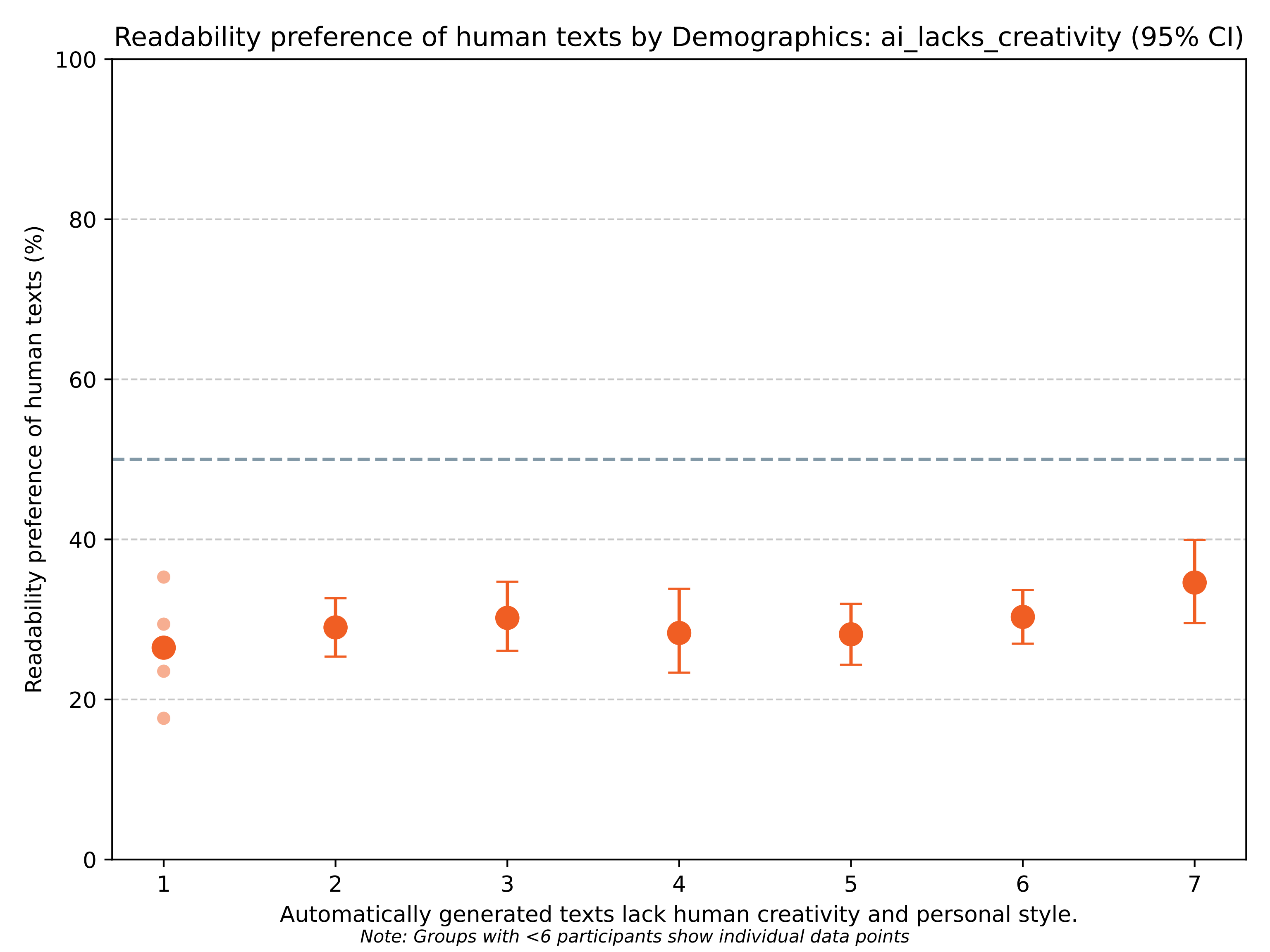

Readability preference of human texts by Demographics: ai_lacks_creativity (95% CI)
Readability preference of human texts (%)
100
80
60
40
20
0
1
2
3
4
5
6
7
Automatically generated texts lack human creativity and personal style.
Note: Groups with <6 participants show individual data points
